# Supplementary material for: Rapid oxygen titration following cardiopulmonary resuscitation mitigates cerebral overperfusion and striatal mitochondrial dysfunction in asphyxiated newborn lambs
Source: J Cereb Blood Flow Metab. 2024 Nov 22;45(4):630–42. doi: 10.1177/0271678X241302738 (PMC11584996; doi:10.1177/0271678X241302738)

**Supplemental File**

## Figure 3B

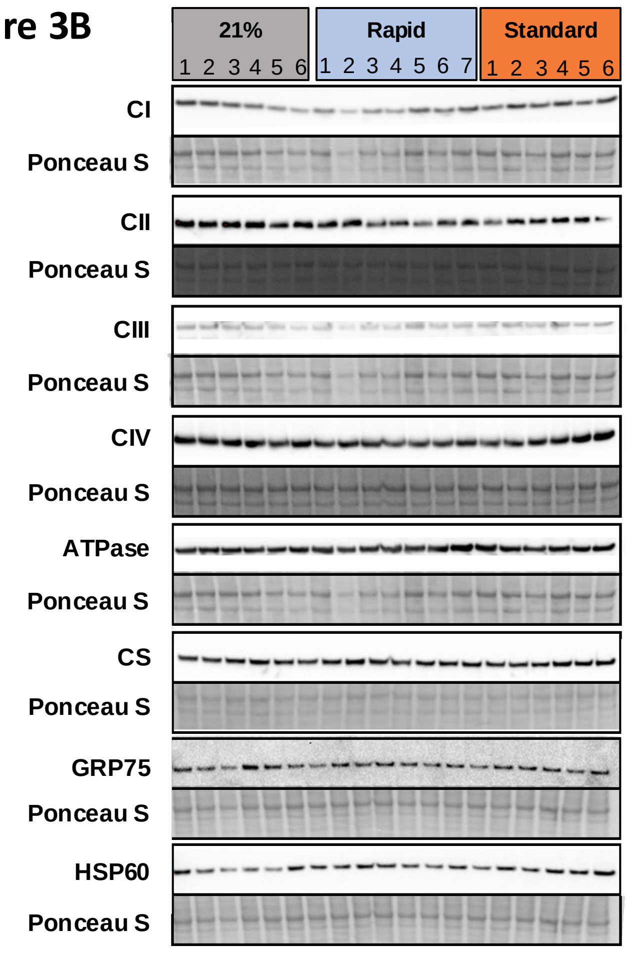


Note: some proteins were probed on the same western blot (CI/CIII/ATPase and GRP75/HSP60).

**Full unedited gel/blot for Figure 3B**

CI

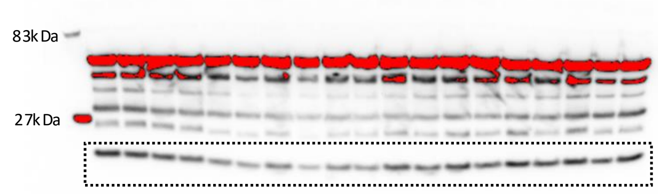


CI

Ponceau S

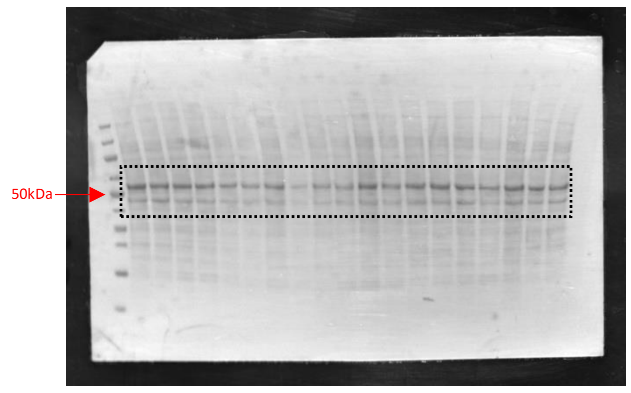


**Full unedited gel/blot for Figure 3B**

CII

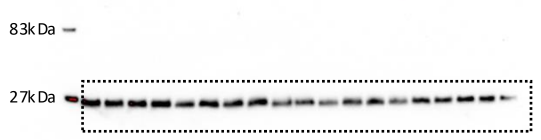


CII

Ponceau S

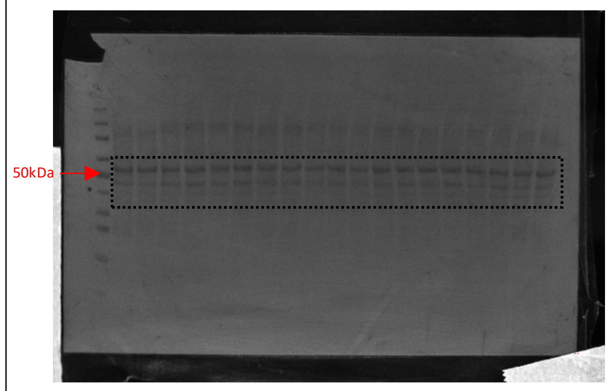


**Full unedited gel/blot for Figure 3B**

CIII

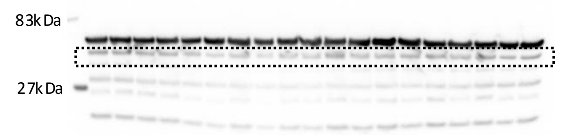


CIII

Ponceau S

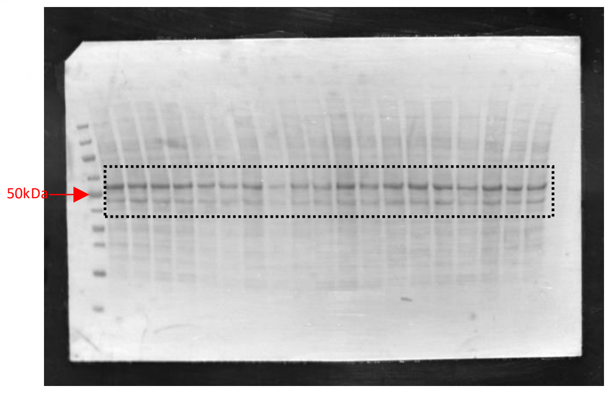


**Full unedited gel/blot for Figure 3B**

CIV

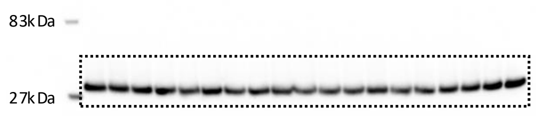


CIV

Ponceau S

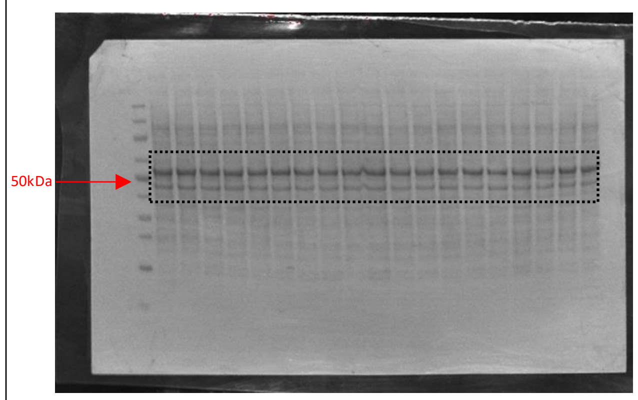


**Full unedited gel/blot for Figure 3B**

ATPase

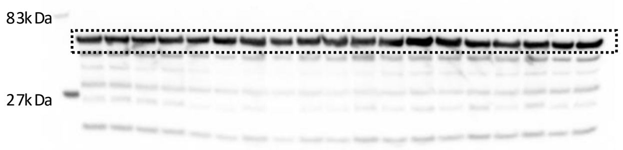


ATPase

Ponceau S

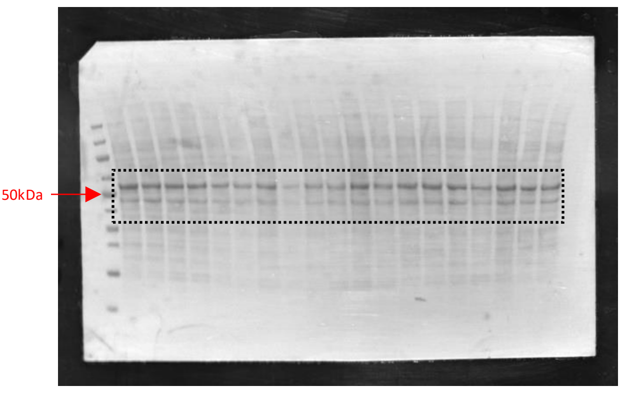


**Full unedited gel/blot for Figure 3B**

CS

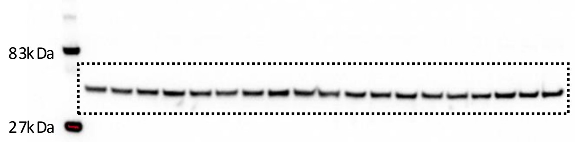


CS

Ponceau S

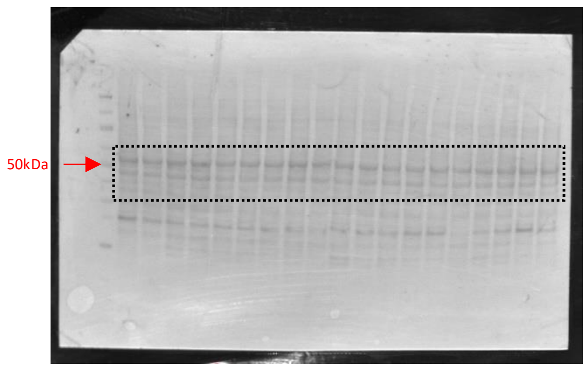


**Full unedited gel/blot for Figure 3B**

GRP75

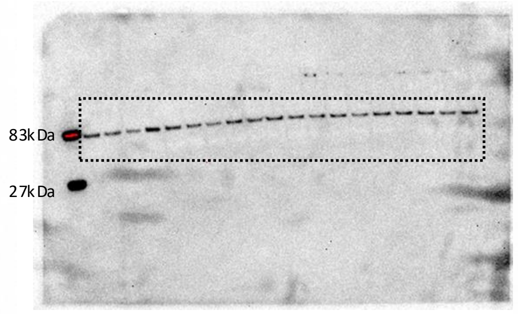


GRP75

Ponceau S

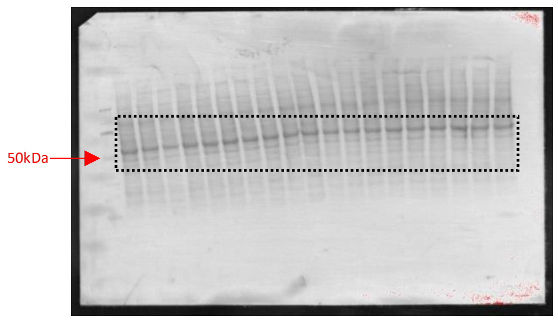


**Full unedited gel/blot for Figure 3B**

HSP60

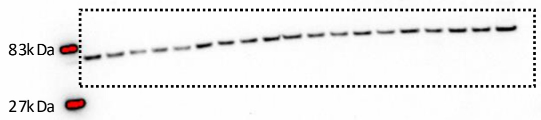


HSP60

Ponceau S

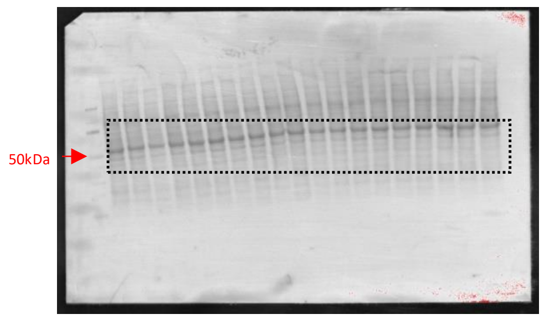

## Supplemental File

### Figure 4B

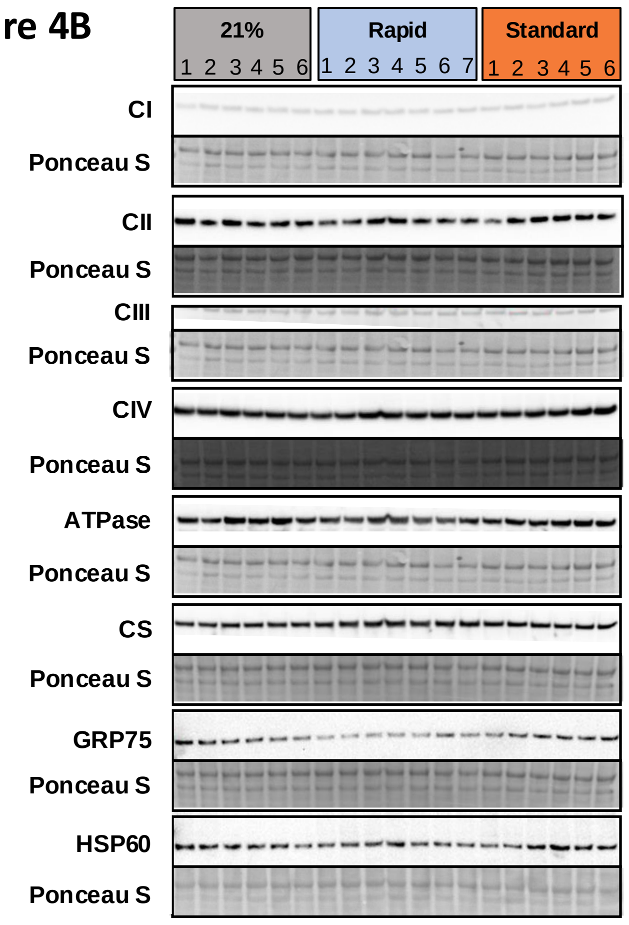


Note: some proteins were probed on the same western blot (CI/CIII/ATPase and GRP75/CS).

**Full unedited gel/blot for Figure 4B**

**CI**

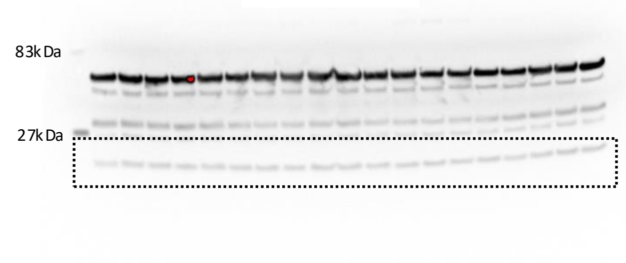


**CI**

**Ponceau S**

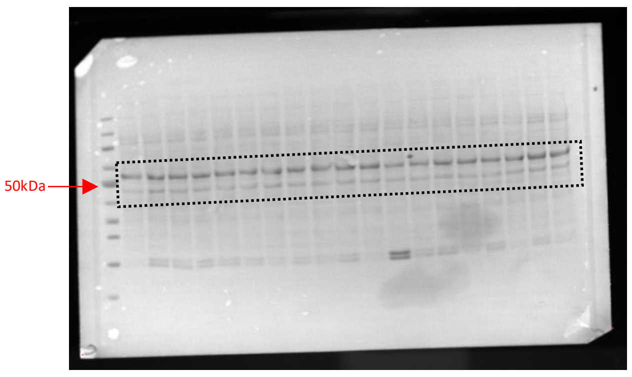


**Full unedited gel/blot for Figure 4B**

**CII**

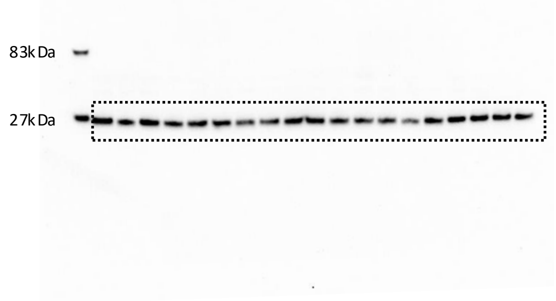


**CII**

**Ponceau S**

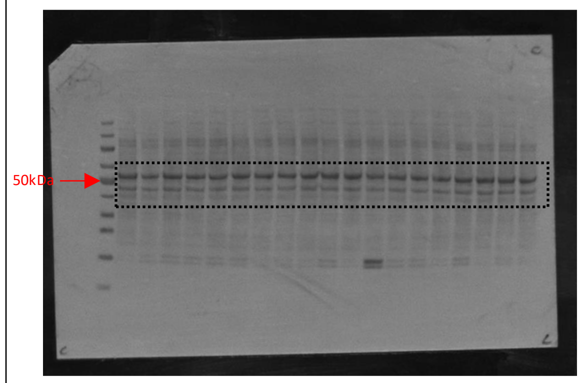


**Full unedited gel/blot for Figure 4B**

**CIII**

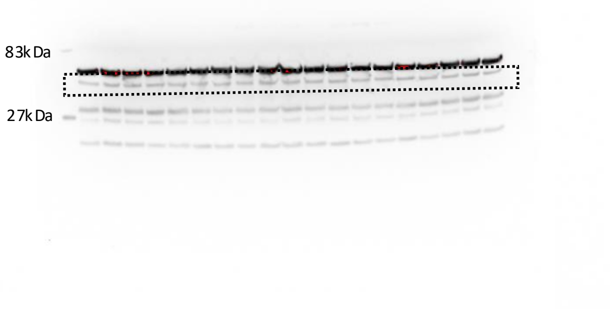


**CIII**

**Ponceau S**

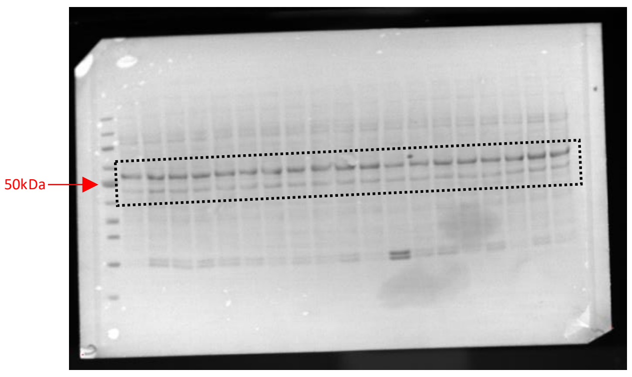


**Full unedited gel/blot for Figure 4B**

**CIV**

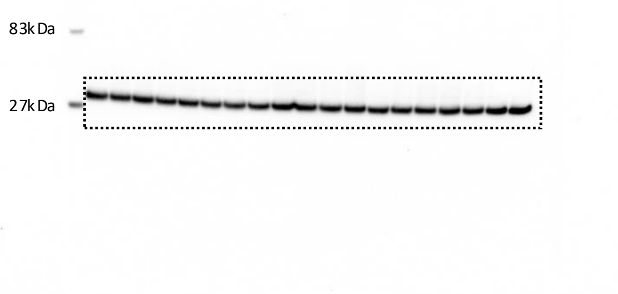


**CIV**

**Ponceau S**

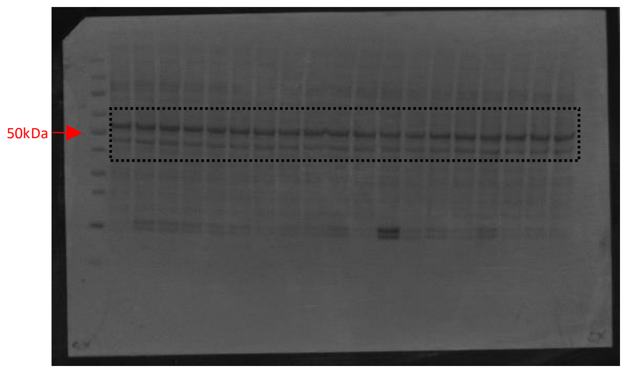


**Full unedited gel/blot for Figure 4B**

**ATPase**

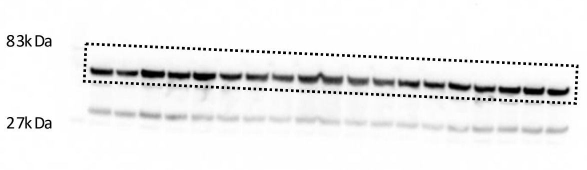


**ATPase**

**Ponceau S**

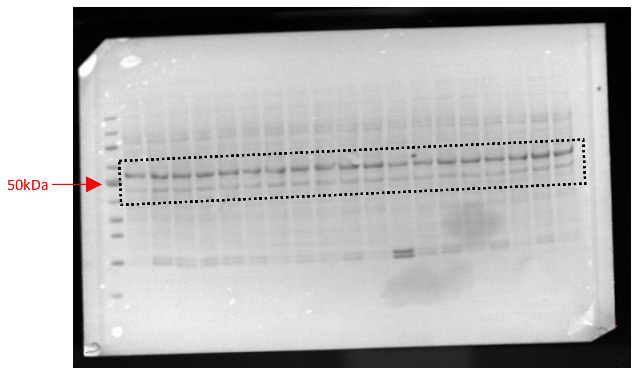


**Full unedited gel/blot for Figure 4B**

**CS**

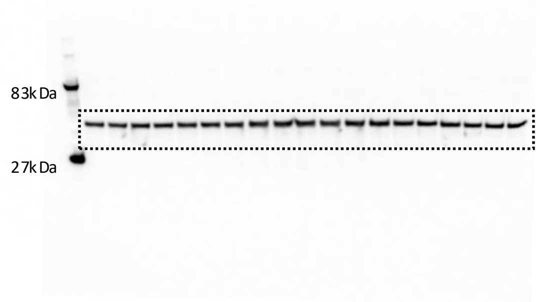


**CS**

**Ponceau S**

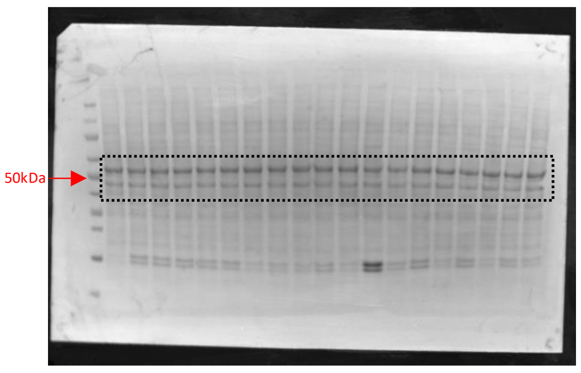


**Full unedited gel/blot for Figure 4B**

**GRP75**

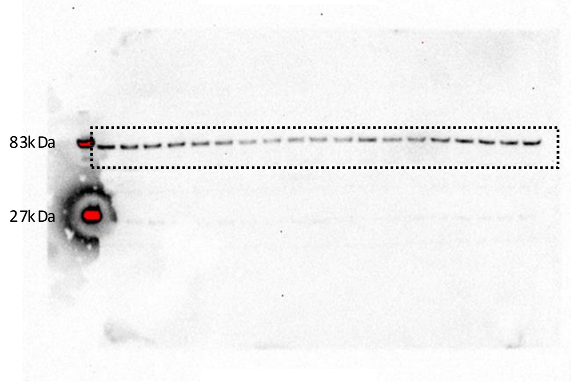


**GRP75**

**Ponceau S**

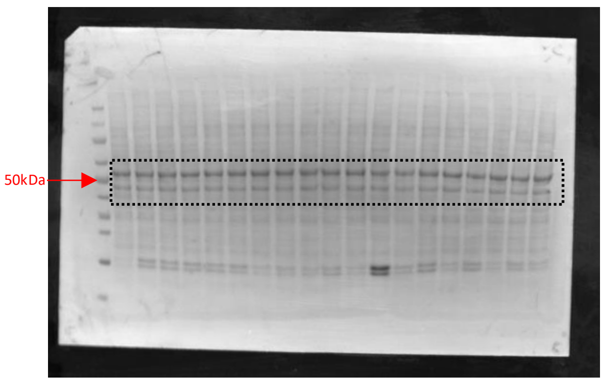


**Full unedited gel/blot for Figure 4B**

**HSP60**

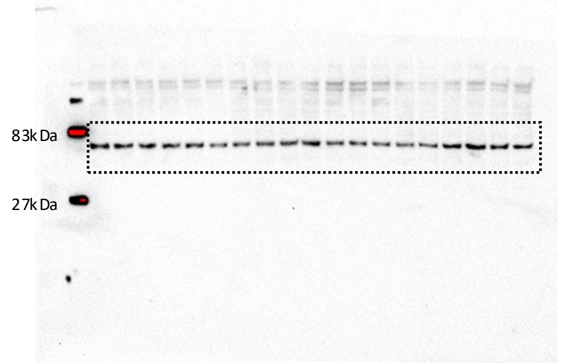


**HSP60**

**Ponceau S**

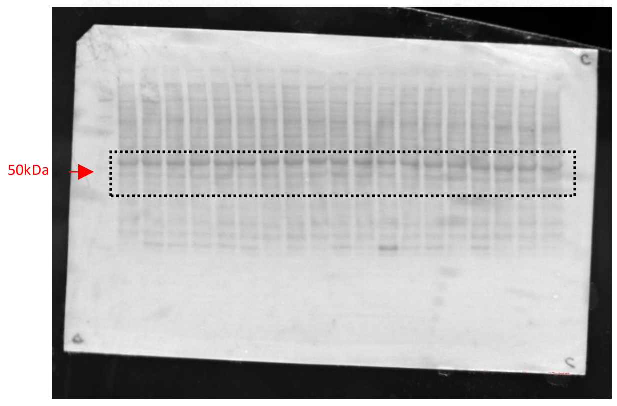

Supplement: sj-pdf-1-jcb-10.1177_0271678X241302738 - Supplemental material for Rapid oxygen titration following cardiopulmonary resuscitation mitigates cerebral overperfusion and striatal mitochondrial dysfunction in asphyxiated newborn lambs [file sj-pdf-1-jcb-10.1177_0271678X241302738.pdf]
